# Comparison of different dosing regimens (once weekly *vs*. twice weekly, and once weekly *vs*. once every two weeks) with epoetin delta in patients with chronic kidney disease: a randomized controlled trial

**DOI:** 10.1186/1745-6215-8-35

**Published:** 2007-11-13

**Authors:** Iain C Macdougall

**Affiliations:** 1 Department of Renal Medicine, King's College Hospital, Denmark Hill, London SE5 9RS, UK

## Abstract

**Background:**

Anaemia is a common complication of chronic kidney disease and prevalence increases with declining renal function. Renal anaemia has significant implications for the well-being and quality of life of patients and impacts on morbidity and mortality. Anaemia can be well managed by therapy with erythropoiesis-stimulating agents (ESAs). Previous clinical trials have shown that the only human cell-line-derived ESA, epoetin delta, is well tolerated and effective in maintaining haemoglobin levels in anaemic patients with chronic kidney disease. The half-life of epoetin delta suggests that administration of this agent is feasible once weekly and once every two weeks. We report on the design and rationale of a trial to compare once weekly *vs*. twice weekly, and once weekly *vs*. once every two weeks dosing of epoetin delta.

**Design and methods:**

This is a randomized, open-label, multicentre trial. Patients aged 18 years or above with chronic kidney disease (Stages 3–5) are eligible to enter this trial. Two groups of patients form the trial population, those naïve to ESA therapy and those previously stable on ESA therapy. There are two primary objectives of this trial: 1) to demonstrate non-inferiority between twice weekly and once weekly dosing of epoetin delta in previously naïve patients (assessed by haemoglobin at Week 24); 2) to demonstrate non-inferiority between once weekly and once every two weeks dosing in previously stable patients (assessed by average haemoglobin over Weeks 16–24). Among the secondary analyses will be assessments of haematocrit, number(%) of patients meeting predefined targets for haemoglobin and haematocrit levels, and comparisons of average dose. All patients will receive study medication for 24 weeks and dose will be adjusted according to a predefined algorithm to achieve and maintain haemoglobin ≥ 11 g/dL. All patients completing this trial are eligible to enter a 2-year follow-up study to enable monitoring of emergent adverse events, anti-erythropoietin antibody responses, maintenance of efficacy and changes in diabetic retinopathy status.

**Discussion:**

To our knowledge, this trial is the first to randomize ESA-naïve patients to different dosing regimens of the same ESA. Data generated will help in guiding the most appropriate dosing frequency for epoetin delta, particularly in those patients new to epoetin delta therapy.

**Trial registration:**

**ClinicalTrials.gov: **NCT00450333

## Background

Chronic kidney disease (CKD) is a growing problem, particularly in the Western world [1] and anaemia is a common complication of CKD, with up to half of all patients affected [2]. Diabetes is the leading cause of CKD in the Western world and in diabetic CKD patients anaemia often develops earlier and more severely than in non-diabetic patients with similar renal function [3,4]. As kidney function declines the prevalence of renal anaemia increases, with most CKD Stage 5 patients (glomerular filtration rate <15 mL/min/1.73 m^2^), on or off dialysis, being anaemic [5]. The primary cause of anaemia in CKD patients is insufficient synthesis of erythropoietin by the damaged kidneys. Anaemia is associated with fatigue and reduced quality of life [6] as well as increased morbidity and mortality [7-9], often due to cardiovascular complications [10]. In addition, anaemia in diabetic patients has been linked with the progression of microvascular complications, including retinopathy [11,12]. Renal anaemia can be managed by treatment with erythropoiesis-stimulating agents (ESAs), which are effective and well-tolerated [8].

The original recombinant erythropoietins (epoetin alfa and epoetin beta) are produced in Chinese hamster ovary cell lines. In contrast, epoetin delta is produced in a human cell line, via a process of gene-activation [13]. Clinical trials have demonstrated that epoetin delta is well tolerated and effective in the management of renal anaemia in CKD patients irrespective of dialysis status or dose frequency [14-17].

In a previous clinical trial epoetin delta administered subcutaneously was shown to be effective in predialysis, peritoneal dialysis and haemodialysis patients [14]. In this trial, epoetin delta administered two- or three-times per week was effective and well tolerated in patients irrespective of dialysis status (predialysis, peritoneal dialysis, haemodialysis). Administration once per week was effective in maintaining haemoglobin levels in predialysis and peritoneal dialysis patients [14,17]. Phase I and II studies of subcutaneous epoetin delta [18] showed that the half-life was similar to that of epoetin beta, an agent that can be administered once per week [19,20]. Patients who are stable on a once weekly dosing regimen with subcutaneous epoetin beta can be switched to once every 2 weeks administration [21]. The combined evidence from clinical trials indicates that it may be possible to extend dosing intervals for epoetin delta beyond once per week. We report on the design and rationale of a trial to assess this potential, highlighting its unique aspects.

## Design and methods

### Design and objectives

This is a multicentre, open-label, randomized, parallel-group study, designed to provide information on extended intervals of dosing with epoetin delta (DYNEPO^®^, Shire plc) in the management of renal anaemia. New dosing regimens will be explored for patients naïve to ESA therapy and patients already stable on ESA treatment.

Two primary objectives have been set for this trial:

1. To demonstrate non-inferiority between twice-weekly (BIW) and once-weekly (QW) dosing of epoetin delta in previously ESA-naïve patients, as assessed by haemoglobin at Week 24

2. To demonstrate non-inferiority between QW and once every two weeks (Q2W) dosing in patients previously stable on an ESA, as assessed by average haemoglobin over Weeks 16–24.

Several secondary objectives, including a key secondary objective, have been included in the trial design and these are detailed below.

Key secondary objective: to demonstrate non-inferiority of efficacy between QW and Q2W dose schedules in patients previously stable on an ESA, as measured by average dose over Weeks 16–24.

Other secondary objectives:

1) To compare efficacy between BIW and QW dose schedules of epoetin delta in previously ESA-naïve patients and between QW and Q2W dose schedules in patients previously stable on an ESA, as measured by:

a) the number (%) of patients who achieve haemoglobin of ≥ 11 g/dL at Weeks 16 and 24 and over Weeks 16–24.

b) haematocrit at Weeks 16 and 24, and over Weeks 16–24.

c) the number (%) of patients achieving the haematocrit target range of 33–36% at Weeks 16 and 24 and over Weeks 16–24.

d) the number (%) of patients who achieve both haemoglobin and haematocrit targets (≥ 11 g/dL and 33–36% respectively) at Weeks 16 and 24 and over Weeks 16–24.

2) To compare the average dose over Weeks 9–16, 16–24 and 9–24 between BIW and QW dose schedules of epoetin delta in previously ESA-naïve patients and between QW and Q2W dose schedules in patients previously stable on an ESA.

3) To compare efficacy between BIW and QW dose schedules of epoetin delta in previously ESA-naïve patients as measured by haemoglobin at Week 16 and over Weeks 16–24.

4) To compare efficacy between QW and Q2W dose schedules in patients previously stable on an ESA as measured by haemoglobin at Weeks 16 and 24.

5) To investigate safety of:

a) BIW and QW dose schedules in previously ESA-naïve patients.

b) QW and Q2W dose schedules in patients previously stable on an ESA.

6) To investigate efficacy and safety of epoetin delta when switching administration frequencies.

7) To summarize efficacy and safety in patients with diabetes mellitus (DM).

### Ethical considerations

This trial will be conducted in agreement with the current applicable regulations, ICH, EU Directive 2001/20/EC, principles of the Declaration of Helsinki and approved by the relevant review board for all participating study centres. All patients will give written informed consent before completing any study-related procedures.

### Study groups

**Figure 1:**
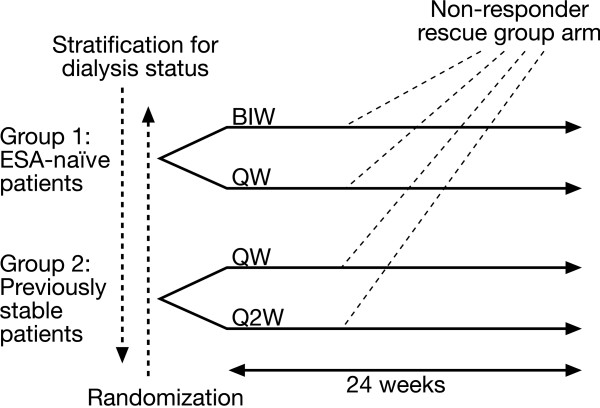
Trial design.

Patients are grouped by previous exposure to an ESA and randomized to differing dose schedules. Group 1 is formed by ESA-naïve patients, Group 2 by patients previously stable on ESA therapy (Figure 1). Patients in both groups are stratified by dialysis status to balance expected adverse event levels. All participants will be ≥ 18 years of age, with anaemia due to CKD (Stages 3–5; GFR < 59 mL./min/1.73 m^2^). The major inclusion and exclusion criteria are shown in Table 1. It is planned to include 208 patients in each arm, randomizing 104 to each dosing regimen, giving a total patient number of 416.

**Table 1:** Major selection criteria

**Inclusion criteria**
• Patients aged at least 18 years of age with CKD (KDOQI Stage 3–5) who are able to give written informed consent and are likely to attend all study visits • Stable on any dose ≤ 10 000 IU/week of subcutaneous ESA or requiring initiation of ESA treatment • Those currently receiving ESA treatment must have been receiving a stable dose, that was effective in managing haemoglobin levels (≥ 11 g/dL), for at least 30 days before randomization in the study • Those requiring initiation of ESA treatment must have haemoglobin levels ≥ 8 g/dL and < 11 g/dL • Transferrin saturation ≥ 20 % and ferritin ≥ 100 ng/mL

**Major exclusion criteria**

• Uncontrolled hypertension • Requiring doses of ESA > 10 000 IU/week • Two or more doses of prescribed ESA treatment missed or withheld by physician order in the 14 days immediately before randomization in the study • Thrombocytopenia (platelet count < 75 000/mm^3^) • Active bleeding disorder (diathesis) (for example, gastro-intestinal or genito-urinary tract bleeding) • Treatment with immunosuppressive drugs (other than corticosteroids for a chronic condition) in the 30 days immediately before randomization in the study • Androgen therapy in the 30 days immediately before randomization in the study • Known HIV infection from medical history • Known or suspected intolerance or hypersensitivity to ESA therapy or to any of the excipients of epoetin delta • Known to have antibodies against erythropoietin • Impaired hepatic function (AST, ALT > 3x ULN)

### Study treatments

All patients will receive epoetin delta, administered subcutaneously, for a maximum of 24 weeks. Dose is adjusted to achieve and maintain haemoglobin ≥ 11 g/dL. If haemoglobin is ≥ 12 g/dL OR the rate of increase of haemoglobin is > 2.5 g/dL/4-week period then the dose is decreased by 25%. If haemoglobin is < 10 g/dL AND the rate of increase is < 0.7 g/dL/4-week period then the dose is increased by 50%. Patients in Group 1 will start on 100 IU/kg/week, and patients in Group 2 will start on a weekly dose of epoetin delta equivalent to the dose of ESA they were previously receiving to maintain stability.

Patients will receive a maximum dose of 20 000 IU at any one time. If the dose schedule determines that they should receive > 20 000 IU they will be withdrawn from their randomized arm and followed for safety in a non-responder rescue group, where their dose of epoetin delta will continue to be adjusted towards a stable dose on a schedule that allows for doses of ≥ 20 000 IU at any one time.

## Procedures

### Enrolment

Patients are participating at sites in Europe and Australasia and enrolment started on 30^th ^October 2006. As of 18th October 2007, 158 patients (43 ESA-naïve) have been enrolled.

### Assessments

The study schedule is shown in Table 2; of note is the assessment of diabetic retinopathy at screening and final visit or withdrawal. This will allow baseline data to be collected in the diabetic participants, who can be followed long-term for changes in retinopathy status.

**Table 2:** Study schedule

**Visit**	**Screening**	**Baseline**	Visit 2*	Visit 3*	Visit 4*	Visit 5*	Visit 6*	Visit 7*	Visit 8*	**Final visit/withdrawal**	**Follow-up call**
**Week**	**Day -14 to -1**	**0**	**2**	**4**	**6**	**8**	**12**	**16**	**20**	**24**	**28**
Informed consent											
In/exclusion criteria											
Randomization											
Demographics											
Medical history											
Physical examination											
Vital signs											
On site assessment of haemoglobin level											
Biochemistry and haematology											
Assay for anti-erythropoietin antibodies											
Echocardiogram											
Retinopathy testing^1^											
Serum pregnancy test											
Urinalysis^2^											
Investigational product dispensed											
Adverse events											
Prior and concomitant medication											
Drug compliance											

*A visit window of ± 3 days will be permitted for study visits 2 to follow-up.

^1^For diabetic patients only.

^2^For patients with CKD stage 3 or 4 only.

### Statistical methods

416 patients will be included in this study (208 naïve and 208 non-naïve). The primary endpoint for this study is haemoglobin (g/dL) at Week 24 for ESA-naïve patients and average haemoglobin over Weeks 16–24 for patients previously stable on an ESA. Based on a similar study [14] and assuming the true difference between dose schedules is zero (using a non-inferiority margin of 0.75 g/dL), 65 patients in each arm are required to complete the trial without major protocol violations to provide 90% power. Full criteria for evaluation are shown in Table 3.

**Table 3:** Criteria for evaluation

**Primary objectives**
• Mean haemoglobin concentration at Week 24 and over Weeks 16–24

**Secondary objectives**

• Number (%) of patients who achieve haemoglobin of ≥ 11 g/dL at Weeks 16 and 24 and over Weeks 16–24 • Mean haematocrit concentration calculated at Weeks 16 and 24 and over Weeks 16–24 • Number (%) of patients who achieve the haematocrit target range of 33–36% at Weeks 16 and 24 and over Weeks 16–24 • Patients' average weekly dose/kg over Weeks 9–16, 16–24 and 9–24 • Mean haemoglobin concentration calculated at Week 16 • Number (%) of patients who achieve both haemoglobin of ≥ 11 g/dL and the haematocrit target range of 33–36% at Weeks 16 and 24 and over Weeks 16–24 • Number (%) of patients with a positive antibody response to epoetin delta • Number (%) of patients shown to have neutralizing antibodies • Blood pressure changes from baseline • Changes in left ventricular ejection fraction from the screening visit • Renal function using estimated glomerular filtration rate • Retinopathy in diabetic patients

ANCOVA analysis will be used for the endpoint of mean haemoglobin at Week 24 in each dose group for the ESA-naïve patients (QW and BIW). A 95% confidence interval (CI) for the difference between QW and BIW in mean haemoglobin will be calculated and non-inferiority of QW compared with BIW will be concluded if the lower limit of the 95% CI lies above -0.75 g/dL. For the endpoint of average haemoglobin over Weeks 16–24 in each dose group for previously stable patients (QW and BIW), repeated measures ANCOVA analysis will be used. A 95% CI for the difference between Q2W and QW in mean haemoglobin will be calculated and non-inferiority of Q2W compared with QW will be concluded if the lower limit of the 95% CI lies above -0.75 g/dL. These results will be assessed in both per-protocol and intent-to-treat populations. All patients who receive study medication will be included in the safety analyses.

### Follow up

All patients completing this study will be eligible for entry into a 2-year, open-label extension to enable monitoring of emergent adverse events, anti-erythropoietin antibody responses, maintenance of efficacy and changes in retinopathy status among the diabetic subpopulation. All patients completing the study, but not entering the open-label extension, will be followed up at Week 28 to review adverse events and record any changes.

## Discussion

Epoetin delta, the first human cell line-derived ESA has been shown to be effective and well-tolerated for the management of anaemia due to CKD, irrespective of dialysis status [14,15]. This trial, designed to assess extended dosing regimens of epoetin delta is the first, to our knowledge, to randomize ESA-naïve patients to different dosing regimens of the same ESA. In addition it addresses the need for a correction phase of sufficient length when commencing ESA therapy. Previous studies have linked anaemia with both increased risk of retinopathy, and in patients with retinopathy, increased risk of progression to severe retinopathy [11]. Monitoring of retinopathy status in the diabetic subpopulation of this trial will provide a patient baseline for assessing the long-term effects of managing anaemia with epoetin delta therapy on this complication of diabetes.

Data generated from this trial should help in offering patients, particularly those new to ESA therapy, flexibility in dosing intervals with epoetin delta. The 2-year follow up to this study will enable long term efficacy and safety data to be collected.

## Competing interests

This study is sponsored by Shire plc. The author is in receipt of a research grant from Shire plc and has acted as a paid consultant to Shire plc.

## Authors' contributions

The author is principal investigator on this trial and contributed to study design and drafting of the manuscript. The author approved the final manuscript.
